# Anti-Ferroptotic Treatment Deteriorates Myocardial Infarction by Inhibiting Angiogenesis and Altering Immune Response

**DOI:** 10.3390/antiox13070769

**Published:** 2024-06-26

**Authors:** Rebecca A. Stairley, Allison M. Trouten, Shuang Li, Patrick L. Roddy, Kristine Y. DeLeon-Pennell, Kyu-Ho Lee, Henry M. Sucov, Chun Liu, Ge Tao

**Affiliations:** 1 Department of Regenerative Medicine and Cell Biology, Medical University of South Carolina, Charleston, SC 29425, USA; stairley@musc.edu (R.A.S.); trouten@musc.edu (A.M.T.); sl178@iu.edu (S.L.); roddyp@musc.edu (P.L.R.); sucov@musc.edu (H.M.S.); 2 Department of Pediatrics, Herman B Wells Center for Pediatric Research, Indiana University School of Medicine, Indianapolis, IN 46202, USA; 3 Division of Cardiology, Department of Medicine, Medical University of South Carolina, Charleston, SC 29425, USA; deleonky@musc.edu; 4 Research Service, Ralph H. Johnson Veterans Affairs Medical Center, Charleston, SC 29401, USA; 5 Department of Medicine Digestive Disease Research Core Center, Medical University of South Carolina, Charleston, SC 29425, USA; leekh@musc.edu; 6 Department of Physiology, Medical College of Wisconsin, Milwaukee, WI 53226, USA; chunliu@mcw.edu

**Keywords:** heart regeneration, myocardial infarction, ferroptosis, angiogenesis, macrophage

## Abstract

Mammalian cardiomyocytes have limited regenerative ability. Cardiac disease, such as congenital heart disease and myocardial infarction, causes an initial loss of cardiomyocytes through regulated cell death (RCD). Understanding the mechanisms that govern RCD in the injured myocardium is crucial for developing therapeutics to promote heart regeneration. We previously reported that ferroptosis, a non-apoptotic and iron-dependent form of RCD, is the main contributor to cardiomyocyte death in the injured heart. To investigate the mechanisms underlying the preference for ferroptosis in cardiomyocytes, we examined the effects of anti-ferroptotic reagents in infarcted mouse hearts. The results revealed that the anti-ferroptotic reagent did not improve neonatal heart regeneration, and further compromised the cardiac function of juvenile hearts. On the other hand, ferroptotic cardiomyocytes played a supportive role during wound healing by releasing pro-angiogenic factors. The inhibition of ferroptosis in the regenerating mouse heart altered the immune and angiogenic responses. Our study provides insights into the preference for ferroptosis over other types of RCD in stressed cardiomyocytes, and guidance for designing anti-cell-death therapies for treating heart disease.

## 1. Introduction

The loss of cardiomyocytes through regulated cell death (RCD) is a key event in the progress of cardiovascular disease [1]. Different forms of cardiac cell death have been implicated in the pathogenesis of various heart diseases, including myocardial infarction (MI), heart failure of diverse etiologies, and congenital heart disease (CHD) [2]. In young hearts, CHDs and chemotherapy-induced cardiomyopathy cause the progressive loss of cardiomyocytes, which leads to impaired heart function [2]. In adults, heart failure caused by MI is one of the main causes of death worldwide [3]. In the infarcted myocardium, ischemia and increased levels of reactive oxygen species (ROS) promote cardiomyocyte death and maladaptive fibrosis [4,5]. Tightly regulated cell death and cellular injury response are crucial for tissue repair and the maintenance of cardiac function [6]. Widely studied forms of RCD in heart disease include apoptosis, necroptosis, and autophagy [2,7,8]. Although cCasp3- (cleaved caspase 3) or TUNEL-positive cardiomyocytes have been observed in animal models of heart attack, the number of apoptotic cells is minimal [9,10]. Our recent work showed that ferroptosis is the major cause of cardiomyocyte loss after MI in neonatal and juvenile mouse hearts [11]. Ferroptosis, a comparatively young type of RCD, is a non-apoptotic form of RCD caused by the excessive production of lipid hydroperoxides in the presence of iron [12]. Ferroptotic cells do not demonstrate apoptotic hallmarks such as caspase activation or cell blebbing [12]. Morphologically, ferroptosis is characterized by shrinkage of the mitochondria and mitochondrial membrane rupture [13,14]. Cellular iron, normally stored in protein complexes composed of ferritins, is required for the onset of ferroptosis [15]. Free iron catalyzes the generation of highly reactive hydroxyl radicals through the Fenton and Haber–Weiss reactions. These hydroxyl radicals promote lipid peroxidation, which is the main cause of ferroptosis [12].

The strong rationale for studying ferroptosis in the heart includes the known cardiotoxicity of iron and the presence of iron overload in the border zone of the infarcted myocardium [16]. In addition, pediatric oncology patients often suffer from cardiac toxicity caused by doxorubicin, a chemotherapy drug effective in treating various cancers [17,18]. Notably, doxorubicin induces oxidative stress and increased levels of labile iron, both of which are key promoters of ferroptosis [19,20].

Two enzymatic proteins have been identified as major inhibitors of ferroptosis. Glutathione peroxidase 4 (Gpx4) inhibits ferroptosis by reducing lipid peroxides to lipid alcohols using its main cofactor, glutathione (GSH) [21]. Meanwhile, ferroptosis suppressor protein 1 (Fsp1) functions as an oxidoreductase on the cell membrane to reduce coenzyme Q10 (CoQ) and generates antioxidants to halt the propagation of lipid peroxidation [22,23]. Recently, increased ferroptosis was observed in adult mouse models of cardiomyopathy [24,25]. Pre-treatment with Ferrostatin-1 (Fer-1) or iron chelator DXZ has been shown to reduce infarct size in adult mouse hearts with ischemic injury [24].

In our recent studies, we found that in young mouse hearts, ischemic cardiomyocytes selectively undergo ferroptosis, instead of apoptosis or necroptosis [11]. We showed that cardiac fibroblasts protect cardiomyocytes from ferroptosis through paracrine effects and direct interaction, highlighting the role of cell–cell interaction in the repair of damaged myocardium [11]. However, it is unclear why cardiomyocytes preferentially undergo ferroptosis after MI, as opposed to other types of RCD. In regenerating myocardium, endothelial cells, macrophages, and fibroblasts participate in tissue remodeling and have a significant impact on the efficiency of regeneration [26]. Angiogenic activity is vital for cardiac muscle repair. Increased angiogenesis promotes the expansion of coronary vasculature into the fibrotic scar that begins as early as 2 days after injury, followed by cardiomyocyte migration [27,28]. Angiogenesis is supported by pro-angiogenic M2 macrophages, which are known to promote endothelial tube formation in vitro through cytokines and growth factors [29]. When macrophages are depleted, the neonatal mouse heart cannot fully regenerate after injury, resulting in increased fibrosis, decreased angiogenesis, and compromised cardiac function [30].

Utilizing mouse heart surgery models and in vitro human iPSC-derived cardiomyocytes, we aimed to address the mechanisms underlying the preference for ferroptosis in cardiomyocytes. We examined the effects of anti-ferroptotic reagents in infarcted young mouse hearts. The result showed that the anti-ferroptotic reagent Fer-1 does not improve heart regeneration and may have deteriorative effects on heart function. In addition, ferroptotic cardiomyocytes play a supportive role during wound healing by releasing pro-angiogenic factors. The inhibition of ferroptosis in the regenerating mouse heart altered the immune and angiogenic responses. Our study shows a positive role of cardiomyocytes undergoing ferroptosis during heart regeneration.

## 2. Materials and Methods

### 2.1. Mouse Strains

All animal protocols and procedures complied with the NIH guidelines and were approved by the Institutional Animal Care and Use Committee (IACUC) of the Medical University of South Carolina (Charleston, SC 29425, USA). *FVB* (JAX 001800) and *C57BL/6J* (JAX 000664) were purchased from the Jackson Laboratory (JAX, Bar Harbor, ME 04609, USA). Male *C57BL/6J* mice were crossbred with female *FVB* mice, and F1 offspring were used for the studies. 

### 2.2. Left Anterior Descending Coronary Artery Occlusion (LAD-O)

For all mouse survival surgeries, littermate controls were used whenever possible. Both male and female mice were distributed randomly among groups. All surgeries were completed with blinding to mouse treatment. Wild-type mice were subjected to LAD-O at P1 or P7 [6]. Briefly, mice were placed under ice to anesthetize them. Nylon sutures (AD Surgical, S-G618R13-U) were used to occlude the left anterior descending artery (LAD). Occlusion was confirmed by the blanching of the myocardium. VetBond tissue adhesive (Santa Cruz Biotechnology, Dallas, TX, USA, NC0846393) was used to close the thoracic cavity. The entire procedure lasted approximately 10 min from hypothermia induction to recovery. Sham procedures were carried out identically without the occlusion of the coronary artery. Ferrostatin-1 (Fer-1, 2 mg/Kg, MilliporeSigma, Burlington, MA, USA, SML0583) or a DMSO vehicle control was administered subcutaneously at 0, 1, 2, and 3 days after surgery. Hearts were collected at 3 days post-surgery for histological examination. For the analysis of cardiac function, mice were subjected to echocardiography 14 days post-surgery; then, hearts were collected for histological examination.

### 2.3. Echocardiography

At 14 days after P1 LAD-O and 21 days after P7 LAD-O, echocardiography was performed using a Vevo 3100 ultrasound system (Fujifilm VisualSonics, Toronto, ON, Canada) equipped with a MX550S transducer. B-mode and M-mode data were acquired following the manufacturer’s guidelines. Each measurement was performed three times per mouse.

### 2.4. Cell Culture

Human umbilical vein endothelial cells (HUVECs, ATCC^®^ CRL-1730™) were cultured in Kaighn’s modification of Ham’s F-12 medium (ATCC, Manassas, VA, USA, 30-2004) and supplemented with 10% FBS (Corning, Corning, NY, USA, 35-011-CV), endothelial cell growth supplement (Corning, Corning, NY, USA, CB-40006), and heparin (Sigma-Aldrich, St. Louis, MO, USA, H4784-250MG). Cells were passaged to passage 3 before being used in tube formation assays.

### 2.5. Differentiation of Human iPSCs to Cardiomyocytes (iCMs)

iPSCs (Cornell Institute for Medical Research, AICS-0048-039) were cultured in mTeSR1media (STEMCELL Technologies, Vancouver, BC, Canada, 85850) on Matrigel (Gibco Thermo Fisher, Waltham, MA, USA, A1413302)-coated plates. At 80% confluency, iPSCs were differentiated into iCMs, as previously described [31]. Briefly, the iPSCs were treated with 8 μM CHIR-99021 (SelleckChem, Houston, TX, USA, S2924) in RPMI (Gibco Thermo Fisher, Waltham, MA, USA, 11875093)-B27(no insulin) (Gibco Thermo Fisher, Waltham, MA, USA, A1895601) from day 0 to 1. Media were changed on day 2 and the cells were treated with 5 μM IWR1 (SelleckChem, Houston, TX, USA, S7086) in RPMI-B27(no insulin) from day 3 to 4. Starting from day 7, RPMI-B27 with insulin (Gibco, Thermo Fisher, Waltham, MA, USA, 17504044) media were given to iPSC-derived cardiomyocytes (iCMs). Two rounds of glucose starvation from day 12 to 15 and from day 20 to 23 were performed to eliminate non-cardiomyocyte cells. At day 30, iCMs were transferred to Matrigel-coated 24-well plates and maintained in RPMI-B27 with insulin for further use.

### 2.6. Conditioned Medium from iCMs

iCMs were transferred to Matrigel-coated 6-well plates, rinsed with PBS, then treated with 30 µM erastin (Sigma-Aldrich, St. Louis, MO, USA, E7781), 1 mM staurosporine (Sigma-Aldrich, St. Louis, MO, USA, S6942), or a DMSO vehicle control diluted in 3 mL of RPMI-B27 with insulin per well. After 6 h, media were removed, and iCMs were rinsed three times with PBS to remove all traces of erastin, staurosporine, or DMSO. Fresh RPMI-B27 with insulin was added to the iCMs for an overnight conditioning period of 16 h. Conditioned media were collected and filtered through a 0.2 µm Supor^®^ membrane (Pall Laboratory, Port Washington, NY, USA, 4612) for cytokine array analysis and HUVEC tube formation experiments.

### 2.7. HUVEC Tube Formation Assay

HUVECs were seeded at a density of 7.5 × 10^4^ cells per well in 24-well plates pre-coated with Matrigel. HUVECs were then treated with conditioned media from iCMs mixed 1:1 with complete HUVEC media for a total volume of 1 mL per well. Cells were incubated for 6 h at 37 °C and 5% CO_2_ and imaged with a Leica DMi1 inverted phase-contrast microscope every hour to document tube formation. Images were analyzed with the Angiogenesis Analyzer plugin from FIJI [32,33]. For our application, the parameters evaluated were the number of junctions, nodes, and extremities, total branching length, total segment length, total mesh area, and mean mesh size. Three independent experiments were performed for each tube formation assay, using at least three wells per condition.

### 2.8. Cytokine Array Assay

Conditioned media of iCM culture were applied to the Proteome Profiler Array Human XL Cytokine Array Kit (R&D Systems, Minneapolis, MN, USA, ARY022B), according to the manufacturer’s instructions. Then, 3 mL of conditioned media from erastin- or DMSO-treated iCMs was concentrated down to 200 μL using MilliporeSigma’s Protein Concentration Kit (Fisher Scientific, Waltham, MA, USA, 50-525-36). An equal amount of total protein from each group (determined by a BCA protein assay) was loaded onto the cytokine array. The blotting signal was visualized in a ChemiDoc Touch Imaging System (Bio-Rad, Hercules, CA, USA, 12003154). Blot analysis was performed using FIJI (NIH), and a heatmap was generated with R-Studio. Gene Ontology analysis was performed using Metascape [34].

### 2.9. Western Blot

Conditioned media were prepared from the iCM culture, as described above. Protein concentration was determined using a Pierce BCA protein assay kit (Thermo Fisher, Waltham, MA, USA, PI23227), according to the manufacturer’s instructions. Western blot was performed as previously described [11]. Total protein was examined using Ponceau S staining solution (Thermo Scientific, Waltham, MA, USA, A40000279) and imaged using the Bio-Rad ChemiDoc Imaging System (Bio-Rad, Hercules, CA, USA, 12003154). Target proteins were detected using SuperSignal West Pico Chemiluminescent Substrate (Thermo Fisher, Waltham, MA, USA, 34577). Target band intensities were quantified using FIJI software (ImageJ2, version: 2.14.0/1.54f National Institutes of Health). The primary antibodies used were IL-19 antibodies (1:500, Bio-techne R&D systems, Minneapolis, MN, USA, AF1035). The secondary antibodies used were donkey-anti-goat horseradish peroxidase (HRP)-conjugated antibodies (1:2500, Bio-techne R&D systems, Minneapolis, MN, USA, HAF109).

### 2.10. Dissociation of Ventricular Cardiac Cells

At 3 days after the P1 LAD-O, the hearts were harvested and rinsed with ice-cold HBSS (Thermo Fisher, Waltham, MA, USA, 14025092) in 60 mm cell culture dishes. The atrial tissue was removed, and the ventricular tissue was cut into 1 mm pieces and incubated in 5 mL of collagenase solution containing 600 U/mL collagenase type II (Thermo Fisher, Waltham, MA, USA, 17101015) and 60 U/mL DNase I (Sigma-Aldrich, St. Louis, MO, USA, AMPD1) for 15 min at 37 °C and 5% CO_2_. The tissue was gently pipetted up and down to facilitate the digestion. The supernatant was collected and stored on ice to quench the collagenase activity. Then, 5 mL of fresh collagenase working solution was added to the remaining heart tissue for a second round of digestion. A total of 5 rounds of digestion were performed. Single-cell suspensions collected at each step were consolidated in their corresponding 15 mL tubes and centrifuged at 300× *g* for 10 min. The supernatant was discarded and the pellet was dissociated with 2 mL of ice-cold FACS buffer (PBS + 1% BSA + 0.5 mM EDTA). The cell suspension was filtered using a 30 μm cell strainer, and then subjected to a 2 min incubation with red blood cell lysis buffer (Miltenyi, Auburn, CA, USA, 130-094-183) followed by centrifugation, resuspension, and cell counting with a hemocytometer. 

### 2.11. Flow Cytometry

Isolated cardiac cells were resuspended in FACS buffer and incubated with FCR blocking reagent (Miltenyi, Auburn, CA, USA, 130-092-575) for 20 min at 4 °C to block the Fc receptor. Following washing with the FACS buffer and resuspension, the cells were stained with Viobility™ 405/520 Fixable Live/Dead Dye (1:1000 dilution, Miltenyi, Auburn, CA, USA, 130-130-404) to exclude dead cells and debris. To prepare single-channel controls for the flow cytometry analysis and compensation, compensation beads (BioLegend, San Diego, CA, USA, 424602) were prepared according to the manufacturer’s protocol and included in the surface-marker antibody staining. To identify macrophages and their subtypes, cells were stained with the following surface-marker antibodies for 20 min at 4 °C: Brilliant Violet 421-conjugated anti-CD206 (rat, 1:20 dilution, BioLegend, San Diego, CA, USA, 141717); Alexa Fluor 488-conjugated anti-F4/80 (rat, 1:50 dilution, Thermo Fisher, Waltham, MA, USA, L34959); PECy7-conjugated anti-CD86 (rat, 1:10 dilution, Miltenyi, Auburn, CA, USA, 130-105-135); and APC-conjugated anti-Ly6C (recombinant, 1:15 dilution, Miltenyi, Auburn, CA, USA, 103-111-779). Flow cytometry was performed using a MACSQuant Analyzer 10 Flow Cytometer, and analyses were performed with FlowJo software v10.9 software (BD Life Sciences, Franklin Lakes, NJ, USA). Doublets were excluded based on FSC-A vs. FSC-H values, and then dead cells were excluded with live/dead dye staining. Lymphocytes were gated based on SSC-A and FSC-A.

### 2.12. Tissue Processing, Histology, and Immunohistochemistry

Hearts were fixed in 10% formalin (VWR, Radnor, PA, USA, 10015-192) at room temperature overnight with continuous rocking. Tissue was then processed for paraffin embedding and sectioned at 7 μm thickness, deparaffinized in xylene, rehydrated, and subjected to histology or immunofluorescent staining. Tissue slides were incubated in humid chambers at 4 °C in primary antibodies overnight in 1% BSA in PBS. On the second morning, slides were washed in PBS, then incubated in the dark at room temperature for 1 h in secondary antibodies in 1% BSA in PBS. Slides were washed with PBS and counterstained with DAPI (Sigma, St. Louis, MO, USA, D9542), then mounted in VECTASHIELD hard-set mounting medium (Vector Laboratories, Newark, CA, USA, H1400). Immunofluorescent images were acquired using a Leica SP8 confocal microscope (Leica Microsystems, Wetzlar, Hesse, Germany). The primary antibodies used included cleaved caspase 3 (1:200, Cell Signaling, Danvers, MA, USA, #9664), cardiac troponin T (cTnT) (1:400, Invitrogen Thermo Fisher, Waltham, MA, USA, #MA5-12960), cTnT (1:400, Fisher, Waltham, MA, USA, #ms-295p1abx), MF20 (1:400, Developmental Studies Hybridoma Bank, Iowa City, IA, USA, #AB_2147781), 4-HNE (1:400, Bioss, Woburn, MA, USA, #bs-6313R), EMCN (1:400, Santa Cruz, Dallas, TX, USA, #sc-65495), PH3 (1:200, Cell Signaling, Danvers, MA, USA, #9701), Mac-3 (1:100, Cedarlane Labs, Burlington, ON, Canada, #CL8943AP), and CD206 (1:200, Bioss, Woburn, MA, USA, #bs-4727R). Secondary antibodies were all used at a dilution of 1:400 and included Thermo Invitrogen #A21200 Chicken anti-mouse IgG 488, Thermo Invitrogen #A-21428 Goat anti-Rabbit IgG (H + L) Cross-Adsorbed Secondary Antibody, Alexa Fluor 555, Thermo Invitrogen #A-21247 Goat anti-Rat IgG (H + L) Cross-Adsorbed Secondary Antibody, Alexa Fluor 647, Thermo Invitrogen #A16034 Donkey anti-rabbit IgG (H + L) TRITC, Thermo Invitrogen #A21082 donkey anti-goat IgG 633, and Thermo Invitrogen  #A31573 donkey anti-rabbit IgG 647. For measuring fibrotic scarring, a Masson’s trichrome staining kit was used (Sigma, St. Louis, MO, USA, HT15). Tissue slides were deparaffinized, rehydrated, and washed with water, before staining in iron hematoxylin working solution for ten minutes. Slides were then rinsed in water, stained in scarlet acid fuchsin, rinsed in water, stained in phosphomolybdic–phosphotungstic acid solution, stained in aniline blue solution, rinsed in water, stained in 1% acetic acid solution, and rinsed in water before proceeding with rapid dehydration and clearing with xylenes. Tissue slides were mounted with Shurmount mounting medium (General Data, Cincinnati, OH, USA, LC-A). 

### 2.13. Single-Cell RNA-Seq Analysis

GEO dataset GSE128628 was used for the scRNA-Seq analysis [35]. Fastq files were aligned to the mm10/GRCm38 reference transcriptome using the count function in the Cell Ranger software (v7.1, 10x Genomics, Pleasanton, CA, USA). The resulting gene expression matrix was further analyzed in R using Seurat (v4.4.0) [36]. Thresholding was used to exclude low-quality cells. Uniform Manifold Approximation and Projection (UMAP) was used for dimensional reduction, and unsupervised clustering was carried out using the RunUMAP and FindClusters functions in Seurat, respectively. Clusters were annotated based on marker gene expression. Cell interactions were inferred and visualized using the package [37].

### 2.14. Statistics

All quantitative experiments included at least 3 biological replicates. Animal studies included at least 3 mice per group. Statistical significance was determined via *t*-tests. Equal variance was determined using Levene’s test. Outliers were determined by Grubb’s test. All statistical work was completed using IBM SPSS Statistics for Macintosh, Version 28.0.1.0. All bar graphs included scattered dots. All bar graphs represent mean ± SD. A *p*-value lower than 0.05 was considered statistically significant.

## 3. Results

### 3.1. Anti-Ferroptotic Treatment Does Not Improve Heart Regeneration after MI

**Figure 1:**
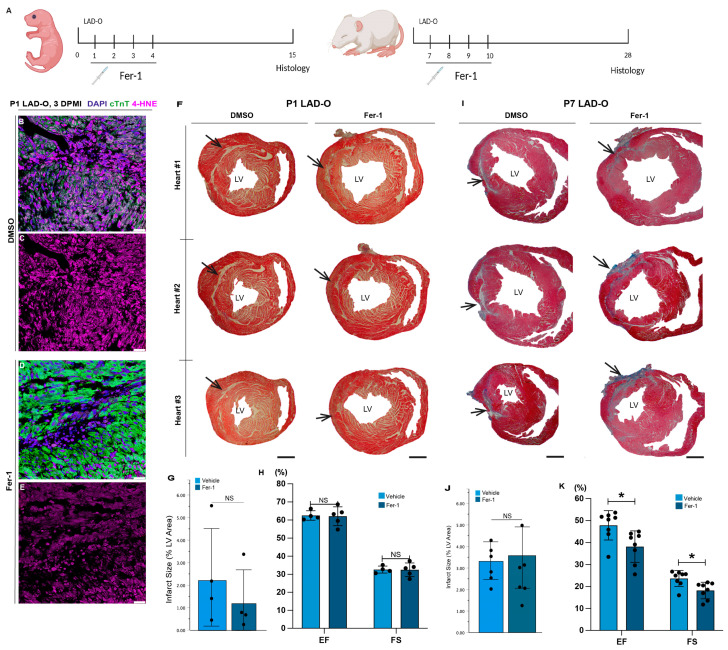
Inhibition of ferroptosis in P1 and P7 hearts after LAD-O does not improve myocardial repair or cardiac function. (**A**) LAD-O was performed at P1 or P7, and Ferrostatin-1 or vehicle control (DMSO) was administered subcutaneously at 0, 1, 2, and 3 days after surgery. Hearts were analyzed at 14 (P1 group) or 21 (P7 group) days after surgery. (**B**–**E**) Tissue sections of infarct zone from DMSO- (**B**,**C**) and Fer-1-treated (**D**,**E**) hearts stained for cTnT (green), 4-HNE (magenta), and DAPI (blue) 3 days after surgery. (**F**–**H**) Heart sections of three representative hearts from control (DMSO) and Fer-1-treated group stained with Masson’s trichrome to show infarcted area in blue (arrows) 14 days after P1 LAD-O. The size of infarct is measured in (**G**). Ejection fraction (EF) and fractional shortening (FS) were measured by echocardiography (**H**). (**I**–**K**) Heart sections of three representative hearts from each group 21 days after P7 LAD-O stained with Masson’s trichrome to show infarcted area in blue (arrows). The size of infarct is measured in (**J**). EF and FS were measured by echocardiography (**K**). *, *p* < 0.05. NS, not significant. Scale bar, 25 μm (**B**–**E**) and 500 μm (**F**,**I**). LV, left ventricle.

To determine if the inhibition of ferroptosis improves cardiac regeneration after myocardial injury, we performed left anterior descending coronary artery occlusion (LAD-O) on wild-type mouse hearts at either the regenerative postnatal day 1 (P1) or the non-regenerative P7 [6]. Mice were treated with Ferrostatin-1 (Fer-1), a synthetic small-molecular hydroperoxyl radical scavenger, or DMSO as the vehicle control (Figure 1A) [38]. Although it requires reduced iron, Fer-1 is not consumed while exerting its antioxidant activity, making it a highly efficient inhibitor of ferroptosis [12,38,39]. Treating infarcted hearts with Fer-1 reduces the level of 4-Hydroxynonenal (4-HNE), a byproduct of lipid peroxidation, in cardiomyocytes (Figure 1B–E) [24]. After P1 LAD-O, while the Masson’s trichrome showed residual scarring in the control group treated with the vehicle (DMSO), as previously reported [6], treatment with the anti-ferroptotic Fer-1 did not further reduce the scarring (Figure 1F,G), nor did it impact the cardiac function, demonstrated by the ejection fraction (EF) and fractional shortening (FS) (Figure 1H). We further investigated the effect of Fer-1 in mouse hearts injured at the non-regenerative stage [40]. After P7 LAD-O, Fer-1-treated and control hearts showed a comparable size of scarring (Figure 1I,J). Interestingly, the assessment of cardiac function using echocardiography showed further decreased EF or FS after Fer-1 treatment, compared to the controls (Figure 1K). These data show an unexpected impact of the anti-ferroptotic reagent on heart regeneration that is against our hypothesis, suggesting a previously unappreciated role of ferroptosis in heart injury.

### 3.2. Fer-1 Treatment Reduces Angiogenesis in Regenerating Hearts

**Figure 2:**
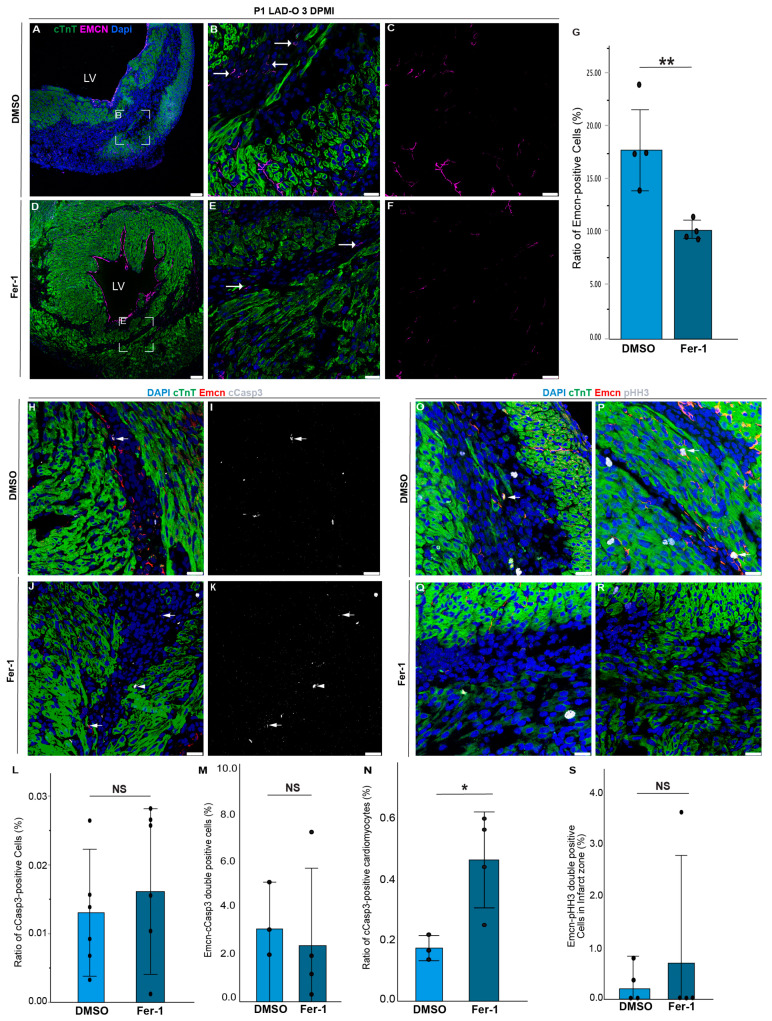
Angiogenesis in infarcted myocardium is inhibited by Fer-1 administration. (**A**–**G**) At 3 days after P1 LAD-O, tissue sections from hearts treated with vehicle control (DMSO, (**A**–**C**)) and Fer-1 (**D**–**F**) were stained for Emcn (magenta) and cTnT (green). White boxes in (**A**) and (**D**) zoom in on (**B**) and (**E**), respectively. Arrows in (**B**,**E**) show Emcn-positive cells. Ratio of Emcn-positive cells is counted in G. (**H**–**L**) Tissue sections from DMSO- (**H**,**I**) and Fer-1-treated (**J**,**K**) hearts were stained for cTnT (green), Emcn (red), and cleaved Caspase 3 (cCasp3, grey). Arrows in (**H**–**K**) show emcn-cCasp3 double-positive cells. Arrowheads in (**J**,**K**) show cCasp3-positive cardiomyocytes. Ratio of total cCasp3-positive cells is quantified in (**L**). Ratio of cCasp3-positive cells in Emcn-positive population is quantified in (**M**). Ratio of cCasp3-positive cardiomyocytes is quantified in (**N**). (**Q**–**S**) Tissue sections from DMSO- (**O**,**P**) and Fer-1-treated (**Q**,**R**) hearts were stained for cTnT (green), Emcn (red), and Phospho-Histone H3 (pHH3, grey). Arrows in (**O**,**P**) show cells positive for Emcn and pHH3. Ratio of total Emcn-pHH3 double-positive cells is quantified in (**S**). Nuclei were stained with DAPI (blue). *, *p* < 0.05. **, *p* < 0.01. NS, not significant. Scale bar, 75 μm (**A**,**D**) and 25 μm (**B**,**C**,**E**,**F**,**H**–**K**,**O**–**R**). LV, left ventricle.

**Figure 3:**
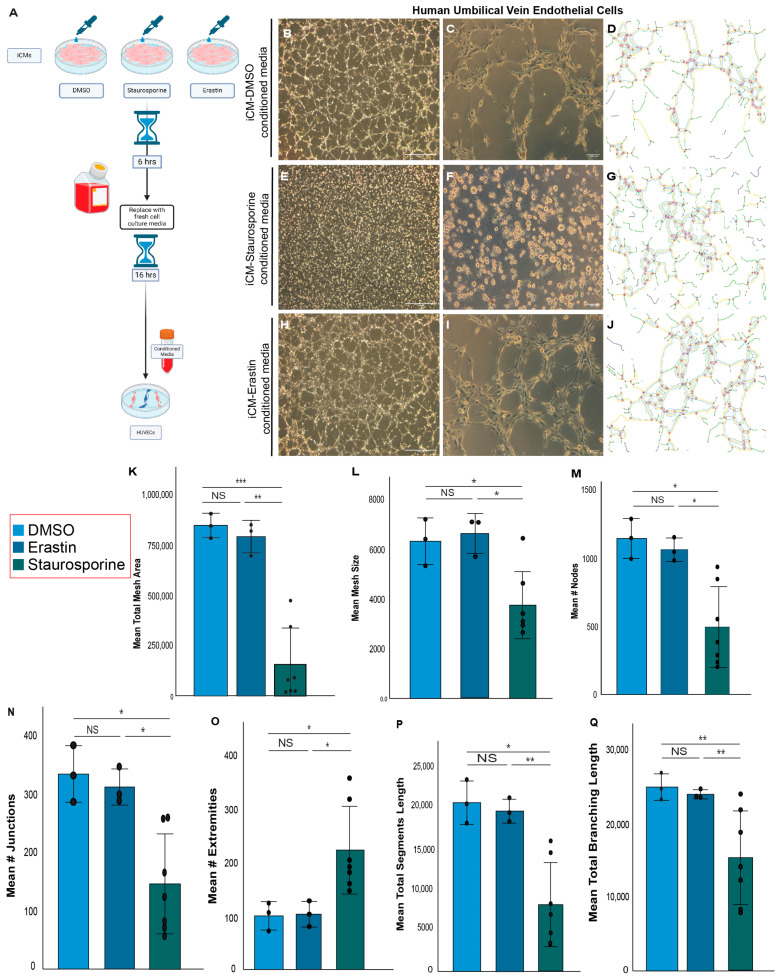
Conditioned media from ferroptotic iCMs support tube formation of HUVECs. (**A**) Schematic plan of production of conditioned media from iCMs. (**B**–**J**) Tube formation of HUVECs cultured for 6 h in conditioned media prepared from iCMs treated with vehicle control (DMSO, **B**–**D**), staurosporine (1 mM, (**E**–**G**)), or erastin (30 μM, (**H**–**J**)). Angiogenesis Analyzer (**D**,**G**,**J**) shows branches (green lines), junctions (blue circles), nodes (red circles), segments (yellow lines), meshes (light-blue lines), close master junctions (purple lines connecting two junctions), extremities (white lines pointing to yellow circles), and isolated segments (dark-blue lines) in (**C**,**F**,**I**), respectively. (**K**–**Q**) Quantification of tube formation metrics. Values are averaged and plotted as the fold change over the DMSO control value. *, *p* < 0.05. **, *p* < 0.01. ***, *p* < 0.001. NS, not significant. Scale bar, 100 μm (**B**,**C**,**E**,**F**,**H**,**I**).

Neonatal heart regeneration requires vital cellular activities, including angiogenesis and the proliferation of key cardiac cell types [27,40]. We previously reported that ferroptosis in cardiomyocytes peaks at three days after LAD-O [11]. Daily Fer-1 treatment starting at the time of surgery significantly reduced the population of vascular endothelial cells marked by endomucin (Emcn) (Figure 2A–G) [41]. The decreased number of endothelial cells was not due to an increased apoptotic rate, as the ratio of either total apoptotic cells or apoptotic endothelial cells was comparable between the control and Fer-1-treated groups (Figure 2H–M). Interestingly, we observed an increased rate of apoptosis in cardiomyocytes after LAD-O and Fer-1 treatment compared to the controls (Figure 2N), suggesting a potential shift from ferroptosis to apoptosis in ischemic cardiomyocytes. We also examined the cell cycle activity in endothelial cells. Although more endothelial cells marked by phospho-Histone H3(pHH3) were observed in the control group, the ratio of proliferative endothelial cells was comparable between the control and Fer-1-treated groups due to the lower number of total endothelial cells caused by Fer-1 treatment (Figure 2O–S). Since the administration of Fer-1 was systemic, we further examined if there is a correlation between the ferroptosis of cardiomyocytes and the angiogenic activity of endothelial cells. The crosstalk between different cardiac cell types is vital for heart regeneration [11]. Therefore, we tested the impact of cardiomyocyte-derived factors on the tube formation of HUVECs (Figure 3A). Conditioned media were prepared from human iPSC-derived cardiomyocytes (iCMs) undergoing erastin-induced ferroptosis or staurosporine-induced apoptosis [42,43], and were compared to the DMSO-treated control group (Figure 3A). Erastin inhibits the activity of the cysteine/glutamate antiporter System Xc-, causing decreased GSH synthesis and Gpx4 activity, and therefore induces ferroptosis [42]. Compared to the controls, the conditioned media from the apoptotic iCMs impaired the tube formation of HUVECs (Figure 3B–G). In comparison, the conditioned media of ferroptotic iCMs still supported tube formation and generated endothelial mesh structures comparable to the control group (Figure 3H–L). Meticulous analysis of the HUVEC tube structure showed comparable segment and branching length, number of nodes, and junctions between the control group and the HUVECs treated with the conditioned media of ferroptotic iCMs (Figure 3M–Q). These parameters were significantly lowered by the conditioned media of apoptotic iCMs, which led to an increased number of extremities in the endothelial mesh, a sign of inefficient tube formation (Figure 3O). These findings support the hypothesis that factors released by ferroptotic cardiomyocytes support a pro-angiogenic microenvironment.

### 3.3. Cytokines Produced by Ferroptotic Cardiomyocytes Have Distinct Roles in Immune Modulation

**Figure 4:**
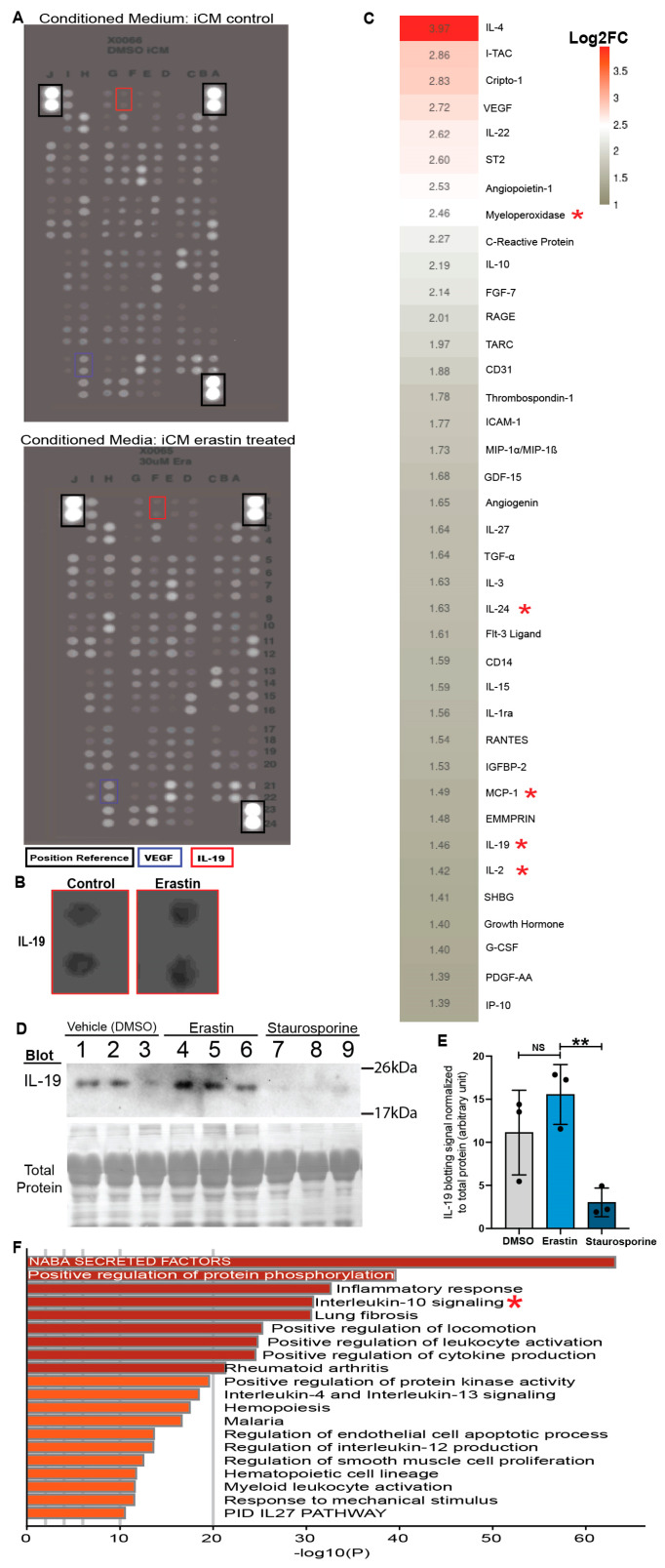
Cytokines and chemokines released by ferroptotic cardiomyocytes have distinct roles in immune modulation. (**A**) Inverted cytokine array blot image of conditioned media from iCMs treated with either vehicle control (DMSO) or erastin (30 μM). (**B**) Zoomed-in images of blotting spots of IL-19, highlighted by red boxes in A. (**C**) Heat map depicting the level of secreted cytokine as log_2_ of fold change (FC) in pixel intensity compared to the control. Red asterisks mark factors associated with Interleukin-10 signaling. (**D**,**E**) Immunoblotting of IL-19 in conditioned medium samples from iCMs treated with vehicle (DMSO), erastin, or staurosporine, *n* = 3 (**D**). Signal intensity of the IL-19 blotting band normalized to total protein (Ponceau) and plotted in (**E**). (**F**) Gene Ontology (Metascape) analysis of cytokines that increased in conditioned media after erastin treatment. **, *p* < 0.01. NS, not significant.

**Figure 5:**
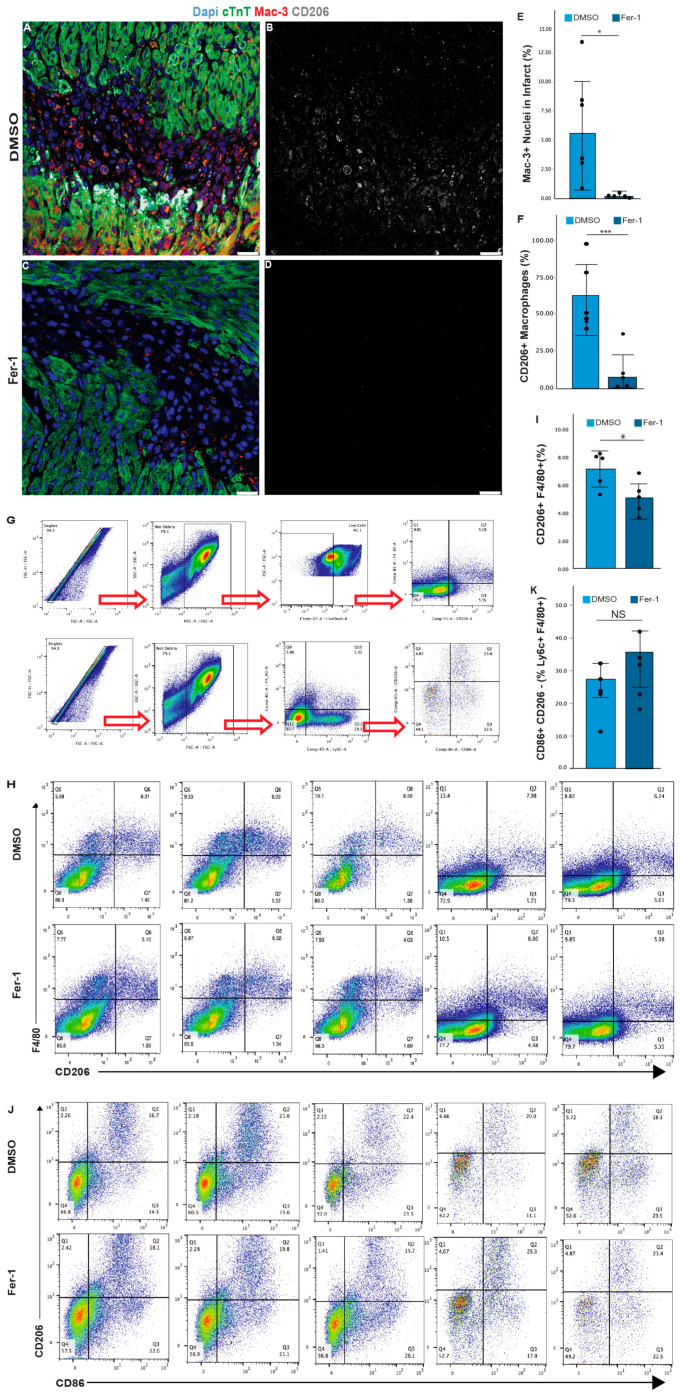
Ferrostatin-1 treatment alters the immune cell population in the infarct zone after LAD-O. (**A**,**C**) Tissue sections from hearts at 3 days after LAD-O treated with vehicle control (DMSO) (**A**,**B**) or Fer-1 (2 mg/kg) (**C**,**D**), stained for DAPI (blue), cTnT (green), Mac-3 (red), and M2 marker CD206 (grey). Channels of CD206 (grey) are presented in (**B**,**D**). (**E**) Ratio of Mac-3-positive cells in the infarct quantified. (**F**) Ratio of CD206-positive cells in the Mac-3-positive cell population. (**G**–**K**) Flow cytometry analysis of ventricular myocardial tissue collected 3 days after P1 LAD-O and either vehicle control (DMSO) or Fer-1 (2 mg/kg) treatment. (**G**) Gating strategy of flow cytometry study. (**H**,**I**) Density plots showing percentages of F4/80^+^CD206^+^ (M2-like) macrophages after DMSO or Fer-1 treatment, with the ratio quantified in (**I**). (**J**,**K**) Density plots showing percentages of the total F480^+^Ly6c^+^ monocyte-derived macrophage population that are CD206^-^CD86^+^ (M1-like) macrophages after DMSO or Fer-1 treatment, with the ratio quantified in K. *, *p* < 0.05. ***, *p* < 0.001. NS, not significant. Scale bar, 25 μm (**A**–**D**).

To investigate the paracrine factors released by ferroptotic cardiomyocytes, conditioned media from ferroptotic and control iCMs were subjected to a cytokine array to examine the protein levels of 105 cytokines and chemokines (Figure 3A and Figure 4A) [11]. Ferroptotic iCMs released an increased number of factors, including VEGF, Interleukin-19 (IL-19), IL-3, IL-22, IL-10, etc. (Figure 4B,C). We performed a conventional Western blot to confirm the secretion of IL-19 by iCMs undergoing different types of RCD. Interestingly, while erastin-treated iCMs showed an increased trend of IL-19 secretion compared to the group of vehicle controls, apoptotic iCMs treated with staurosporine produced a significantly lower level of IL-19 (Figure 4D,E). This suggests a unique pattern of secretome from ferroptotic cardiomyocytes. Gene Ontology (GO) analysis revealed enrichment in the inflammatory response, the regulation of the endothelial cell apoptotic process, and Interleukin-10 signaling (Figure 4F). Immune cells, including certain subtypes of macrophages, are well known as crucial regulators of angiogenesis [44,45,46]. Meanwhile, macrophages are required for neonatal mouse heart regeneration after MI [30]. Cardiac macrophages can be broadly categorized into the pro-inflammatory M1 and anti-inflammatory M2 groups [47]. We examined the size of the macrophage population when regenerating neonatal hearts with Fer-1 or the vehicle treatment. At 3 days after P1 LAD-O, Fer-1 treatment resulted in a decreased population (from 5.5% to 0.16%) of total macrophages in the infarct zone, as shown by the immunofluorescence of Mac-3, a pan-macrophage marker (Figure 5A–E) [47,48]. The immunostaining of CD206 further showed that the number of M2-like macrophages also decreased dramatically (from 62.51% to 7.94%) in the Fer-1-treated myocardium (Figure 5A–D,F) [47,49,50]. This finding was confirmed by the flow cytometry of macrophages from the regenerating myocardium with Fer-1 or the vehicle treatment (Figure 5G). The F4/80 + CD206+ M2-like macrophages were significantly decreased in the Fer-1-treated hearts (Figure 5H,I) [47]. On the other hand, the Ly6C + F4/80 + CD86 + CD206- M1-like macrophages showed a trend of elevation after Fer-1 treatment, although this was not statistically significant (Figure 5J,K). These findings support the hypothesis that ferroptotic cardiomyocytes polarize the macrophage population towards a pro-regeneration/anti-inflammatory M1 status.

### 3.4. IL-19 Facilitates Myocardial Repair after LAD-O

**Figure 6:**
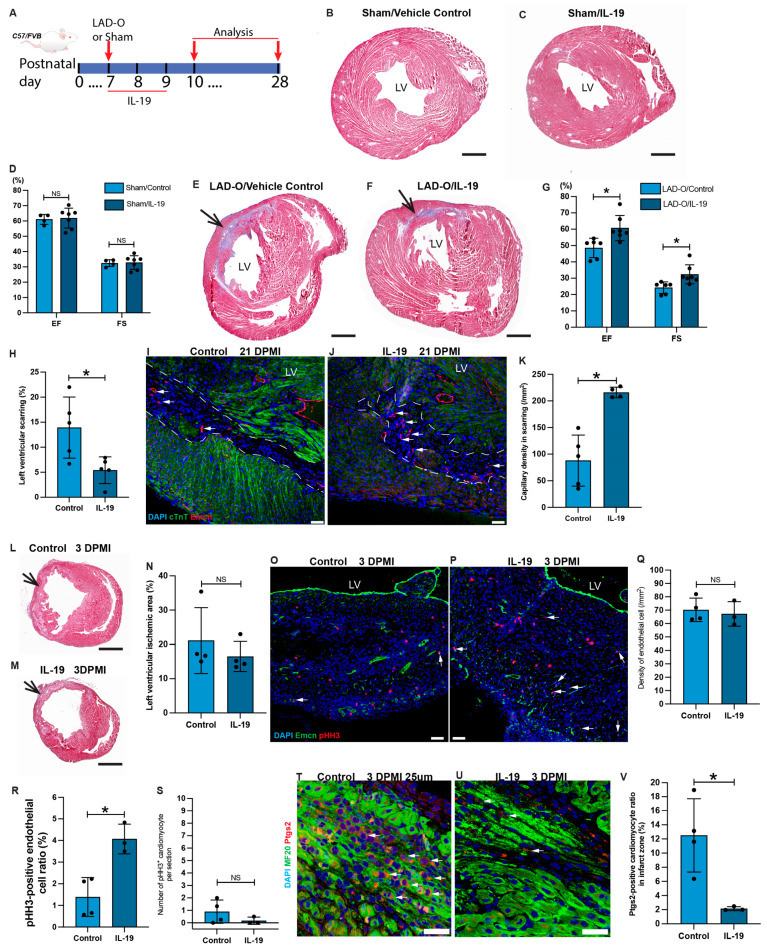
IL-19 promotes cardiomyocyte survival and regeneration after MI. (**A**) Schematic plan for panel B to V. (**B**–**D**) Trichrome of heart sections at 21 days after sham procedure with vehicle control (saline, B) or IL-19 (**C**) treatment. Ejection fraction (EF) and fractional shorting (FS) of vehicle control and IL-19-treated mice were measured and are plotted in (**D**). (**E**–**H**) Trichrome of heart sections at 21 days after LAD-O with vehicle control (**E**) or IL-19 (**F**) treatment. EF and FS of vehicle control and IL-19-treated mice were measured and are plotted in (**G**). Ratio of scarring area over total left ventricular area is quantified in (**H**). (**I**–**K**) Heart sections at 21 days after LAD-O with vehicle control (**I**) or IL-19 (**J**) treatment were stained for cTnT (green) and Emcn (red). Dotted lines encircle the scarring in the left ventricle. Density of capillaries in scar zone is quantified in (**K**). (**L**–**N**) Trichrome of heart sections at 3 days after LAD-O with vehicle control (**L**) or IL-19 (**M**) treatment. Ratio of ischemic zone in left ventricle was measured and is plotted in (**N**). (**O**–**S**) Heart sections at 3 days after LAD-O with vehicle control (**O**) or IL-19 (**P**) treatment were stained for Emcn (green) and pHH3 (red). Density of endothelial cells in scar zone is quantified in (**Q**). Ratio of pHH3-positive endothelial cells is quantified in (**R**). Number of pHH3-positive cardiomyocytes (marked by cTnT, channel not presented) on each tissue section is counted in (**S**). (**T**–**V**) Heart sections at 3 days after LAD-O with vehicle control (**T**) or IL-19 (**U**) treatment were stained for MF20 (green) and Ptgs2 (red). Ratio of Ptgs2-positive cardiomyocytes in scar zone is quantified in (**V**). Nuclei stained with DAPI (blue). All bar graphs represent mean ± SD. *, *p* < 0.05. NS, not significant. Scale bar, 25 μm (**T**,**U**), 50 μm (**I**,**J**,**O**,**P**), and 500 μm (**B**,**C**,**E**,**F**,**L**,**M**). LV, left ventricle. DPMI, days post-MI.

The cytokine array study and the following validation using Western blot identified IL-19 as one of the top hits with increased levels in the conditioned media of ferroptotic iCMs (Figure 4A–E). IL-19 is anti-inflammatory and has been reported to be pro-angiogenic in injured skeletal muscle [51,52]. To determine the roles of IL-19 in heart repair, we treated wild-type mice with IL-19 or a vehicle control (saline) at P7 (non-regenerative stage) after LAD-O or a sham procedure (Figure 6A). IL-19 treatment alone does not affect myocardial structure and function (Figure 6B–D). After LAD-O, the administration of IL-19 preserved cardiac structure and contractility at 21 days after surgery compared to the vehicle controls (Figure 6E–G), and significantly decreased scarring in the infarct zone (Figure 6H). Since IL-19 is a pro-angiogenic chemokine, we examined the density of capillaries in the infarct zone. The immunofluorescence of Emcn showed an increased density of capillaries in the infarcted myocardium with IL-19 treatment (Figure 6I–K). To better dissect the roles of IL-19 in myocardial remodeling and coronary angiogenesis, we examined heart tissue 3 days after LAD-O (DPMI), an acute injury period commonly used in previous studies [6]. The treatment with IL-19 did not alter the area of the ischemic myocardium (Figure 6L–N). The immunofluorescence of Emcn showed a comparable density of capillary endothelial cells in the infarct zone of IL-19-treated hearts, compared to the controls (Figure 6O–Q). However, IL-19 treatment promoted the cell-cycle activity in endothelial cells, as shown by the staining of phospho-Histone H3 (pHH3) (Figure 6O,P,R), supporting the hypothesis that IL-19 promotes myocardial repair through its pro-angiogenic and mitogenic effects on the endothelial cells [53]. Importantly, the beneficial effect of IL-19 is mainly through its angiogenic activity, as the cell-cycle activity in the cardiomyocytes was not altered by the IL-19 treatment (Figure 6S). Interestingly, although ferroptotic cardiomyocytes are a key source of IL-19 after myocardial injury, the administration of extrinsic IL-19 seems to inhibit the spreading of ferroptosis in cardiomyocytes located in the border zone (Figure 6T–V).

### 3.5. Single-Cell RNA-Seq Reveals Interactions between Cardiomyocytes, Macrophages, and Endothelial Cells in Injured Myocardium

**Figure 7:**
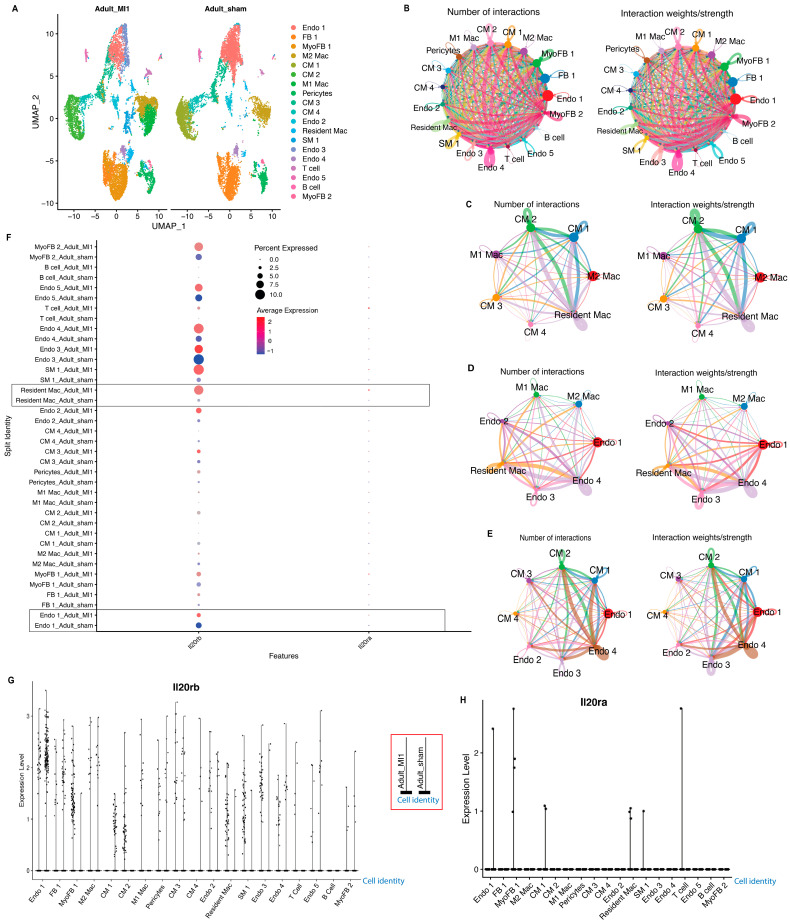
Single-cell RNA-Seq reveals interactions between cardiomyocytes, macrophages, and endothelial cells. (**A**) UMAP showing the 19 color-coded cell clusters based on single-cell RNA sequencing data on adult mouse hearts at 5 days after LAD-O (Adult_MI1) or sham (Adult_sham) procedure (GSE128628). (**B**–**E**) Circle plots depicting the number and strength of the interactions between various cell clusters in the infarcted hearts. (**F**) Dot plot depicting the expression of IL20Rβ and IL20Rα. Boxes highlight the resident cardiac macrophage and Endo 1 populations that show the greatest upregulation in IL20Rβ receptor subunits in response to MI. (**G**,**H**) Feature plot showing the RNA expression levels of each IL20 receptor subunit in each of the 19 cell clusters. Endo, endothelial cell. FB, fibroblast. Mac, macrophage. MyoFB, myofibroblast. CM, cardiomyocyte. SM, smooth muscle. Red box: legend for panels G and H, showing sample identity of each column for every cell identity.

Based on our finding that ferroptotic cardiomyocytes secrete IL-19, which regulates angiogenesis, we hypothesized that IL-19 plays a role in the establishment of cell–cell interaction between cardiomyocytes and endothelial cells. Using a publicly available single-cell RNA-Seq dataset of mouse heart tissue after the LAD-O or sham procedure, we examined the interactions among various cardiac cell types in response to ischemic injury (Figure 7A) [35]. Nineteen distinct types of cell clusters were identified in both the LAD-O and sham hearts (Figure 7A). The circle plots are generated based on RNA expression data, where the size of the circle corresponding to each cell cluster is directly proportional to the number of cells in that cluster, and the width of the line connecting two cell clusters is directly proportional to calculated interactions between the two clusters [54]. The prediction of interactions between each pair of cardiac cell clusters showed strong interactions among subpopulations of cardiomyocytes, endothelial cells, and macrophages (Figure 7B–E). Both cardiomyocytes and cardiac endothelial cells were predicted to establish strong correlation with the cardiac resident macrophages after myocardial injury (Figure 7C,D). The IL-19 signals through the heterodimer receptors comprised IL20Rβ and IL20Rα [51]. Among them, IL20Rβ is strongly expressed in sub-populations of endothelial cells, cardiomyocytes, and macrophages, suggesting that IL-19 derived from ferroptotic cardiomyocytes participates in the intercellular crosstalk among these cell populations (Figure 7F–H).

## 4. Discussion

The mouse heart has a transient regenerative capacity in the neonatal stage (P1) that is gradually lost during cardiomyocyte maturation [26,55,56]. Studies of this early regenerative window have identified several factors that can promote adult mouse heart repair [57]. Neonatal hearts are regenerative due to the residual cell cycle activity of cardiomyocytes [26]. Within days of birth, cardiomyocytes exit the cell cycle, driven by the increased metabolic rate and intracellular oxidative stress [5,6]. Promoting cardiomyocyte cell cycle re-entry for therapeutic purposes has been one of the main foci of the field [58]. However, we would like to raise attention to the notion that cardiomyocyte death and survival are also crucial aspects of heart regeneration.

We recently reported that, after P1 or P7 LAD-O, cardiomyocytes primarily undergo ferroptosis, rather than apoptosis or necroptosis [11]. Due to high energy demand, cardiomyocytes have internal storage for fatty acids and consistently deal with oxidative stress [5]. Both factors are required for the onset of ferroptosis [12]. Ferroptotic cells maintain the ability to regenerate ATP, a feature that seems to be favorable for preserving basic cardiac function in a damaged myocardium [12]. When originally reported, ferroptotic cells were thought to have intact cell membranes, which can limit the number of secretory factors released from stressed cells [12]. However, later evidence showed that this phenomenon could be cell-type specific [59]. Therefore, the status of membranous structures in injured cardiomyocytes will need further study. Moreover, an interesting observation of ferroptosis being reversible was made in vitro [60], supporting the hypothesis that ferroptotic cells undergo an extensive period before reaching the “point-of-no-return” [61]. This could be a required feature of RCD in cardiomyocytes due to their low turnover rate after injury. All the evidence suggests that ferroptosis plays a vital role in the injury response and repair of damaged myocardial tissue. Here, we showed that the inhibition of ferroptosis using a pharmaceutical approach further compromised cardiac function after LAD-O due to decreased angiogenic activity and an altered macrophage population. Secreted factors from the ferroptotic cardiomyocytes, including IL-19, participated in tissue repair after MI.

RCD eliminates damaged cells and prepares the tissue for regeneration. The roles of the to-be-disposed cells vary in different tissue types in relation to different intrinsic regenerative capacities and the preferred type of RCD [61]. The role of cell death has not been appreciated in tissue repair until recent years. Previous studies reported an apoptosis-induced compensatory proliferation in wound healing [62]. As the first member of the RCD family, apoptotic signaling has been shown to trigger cellular remodeling and regeneration in model species, including *Hydra*, zebrafish, and mice [62]. The progression of RCD is highly dynamic and the status of a cell undergoing RCD constantly changes. To identify the factors released by dying cells into the microenvironment, iPSC-derived cardiomyocytes were treated with different reagents at optimized dosages to mimic the stress and dying stages of ferroptosis and apoptosis. Our current study questioned the necessity of suppressing RCD for therapeutic purposes. In cardiac ischemia/reperfusion injury, the priority is to eliminate the pathological cause of the ischemia using techniques such as angioplasty. After that, halting RCD to preserve the remaining cardiomyocytes is likely beneficial for heart function. However, when the tissue ischemia cannot be ameliorated immediately, the suppression of a certain type of RCD might push the stressed cells toward alternative RCD pathways, or even necrosis. Should this happen, excessive inflammation and a lack of RCD-derived signaling for tissue repair may cause more harm than good. In our model, the treatment of anti-ferroptotic Fer-1 caused a potential shift from ferroptosis to apoptosis in ischemic cardiomyocytes, supporting our hypothesis.

Cell–cell interactions between different cardiac cell types are important aspects of heart regeneration. Cardiac fibroblasts showed resistance to ferroptosis and can protect cardiomyocytes from ferroptosis through paracrine signaling and cell–cell contact [11]. The fibrogenic activity of CFs was kept under control by cardiomyocyte-derived Pitx2 signaling [11]. In the current study, we unveiled the regulatory roles of secretory factors derived from ferroptotic cardiomyocytes in macrophage polarization and angiogenesis. The “resident” cardiac macrophages, which are considered pro-regenerative and anti-inflammatory, are quickly depleted after injury [50,63] and replaced by monocyte-derived “infiltrating” macrophages [47]. Macrophages can also be categorized into the pro-inflammatory M1 and pro-regenerative M2 subtypes. The distinction is very nuanced and would be better described as a gradient rather than a binary classification [47]. Therefore, it is more accurate to refer to these macrophages as either M1-like or M2-like. The polarization of macrophages is regulated by chemokines such as the interleukin family. The anti-inflammatory IL-19 belongs to the IL-10 subfamily [64]. Unlike other IL-10 family members, IL-19 is expressed in angiogenic tissue, and has potent pro-angiogenic effects on multiple human endothelial cells through autocrine effects [53]. Our current study showed that healthy iCMs secrete a basal level of IL-19, and that ferroptosis-inducing reagents, but not apoptosis-inducing reagents, promote the secretion of IL-19 from cardiomyocytes. We also showed that IL-19 is chemotactic and mitogenic for cardiac endothelial cells. The administration of extrinsic IL-19 benefits heart repair after MI. Interestingly, IL-19 promotes M2-like macrophage polarization, matching our observation of decreased M2-like population after Fer-1 treatment. Together, these findings implicate IL-19 as a link between anti-inflammation and angiogenesis during heart regeneration, and we further predict the interaction among different cardiac cell types through the IL-19 pathway based on ligand and receptor expression data.

Some limitations of the current study include the fact that IL-19 was not the only factor released by the ferroptotic cardiomyocytes. Other factors, including the anti-angiogenic Tsp-1, also changed during ferroptosis [65]. Therefore, it is important to understand that a stoichiometric pattern of secretome decides the final readout of the regenerative effect and may increase the complexity of the research. Future studies will cover other chemokines released by ferroptotic cardiomyocytes, including IL-33, which induces mitochondrial uncoupling in macrophages to limit ROS and prevent polarization towards the M1 phenotype [66]. In addition, it should be noted that CD206 immunofluorescence is reportedly unable to distinguish between pre-MI resident cardiac macrophages and post-MI M2 macrophages [47]. Clinically, the current study is relevant to pediatric heart disease with cardiomyocyte loss, such as that found in doxorubicin-induced cardiotoxicity. However, it is important for future studies to involve an adult heart injury model to investigate the roles of ferroptosis in mature myocardium.

In summary, our previous and current works suggest that cardiomyocytes preferably undergo ferroptosis to facilitate tissue repair. Anti-ferroptotic reagents should be used wisely for treating heart disease. Ferroptotic cardiomyocytes play a supportive role during wound healing by releasing pro-angiogenic factors. The inhibition of ferroptosis in the regenerating mouse heart altered the immune effect and angiogenic response.

## Data Availability

Data are contained within the article. This paper does not report original sequencing data or code. Single-cell RNA-seq data used were available in GEO (GSE128628). Microscopy data reported in this paper will be shared by the lead contact upon request. Any additional information required to reanalyze the data reported in this paper is available from the lead contact upon request.
